# Protective effects of a probiotic-fermented germinated grain complex on neurotransmitters and sleep quality in sleep-deprived mice

**DOI:** 10.3389/fmicb.2024.1438928

**Published:** 2024-07-29

**Authors:** Jiahua Cheng, Qiqi Wu, Rui Sun, Wujuan Li, Zhuoling Wang, Min Zhou, Tian Yang, Jing Wang, Yuhong Lyu, Changwu Yue

**Affiliations:** ^1^Yan’an Key Laboratory of Microbial Drug Innovation and Transformation, School of Basic Medicine, Yan’an University, Yan’an, China; ^2^Clinical Laboratory, Xi’an Daxing Hospital, Xi’an, China; ^3^Department of Obstetrics and Gynecology, Affiliated Hospital of Yan’an University, Yan’an, China; ^4^Shaanxi Engineering and Technological Research Center for Conversation and Utilization of Regional Biological Resources, Yan’an University, Yan’an, China

**Keywords:** PCPA, sleep deprivation, compound fermentation, neurotransmitters, the microbiota-gut-brain axis

## Abstract

**Objective:**

To explore the effects of probiotic fermentation products of germinated grains on cognitive and sleep improvement in mice with sleep deprivation induced by chlorophenylalanine (PCPA), and to provide theoretical and experimental basis for the development of natural products to alleviate insomnia.

**Methods:**

ELISA and high-performance liquid chromatography (HPLC) were used to determine the contents of γ-aminobutyric acid and L-theanine in fermentation products. Open Field Test was used to analyze the changes of emotional behavior between groups before and after intervention. ELISA was used to analyze the changes of hypothalamic serotonin, GABA, glutamate, and serum interleukin 6. 16S rRNA sequencing was used to analyze the changes of intestinal flora before and after the intervention of compound fermentation products. LC–MS/MS was used to analyze the changes of intestinal SCFAs before and after the intervention.

**Results:**

The content of GABA and L-theanine in 7 L fermentation products was 12.555 μmol/L (1.295 mg/L) and 0.471 mg/mL by ELISA. Compared with the PCPA-induced Model group, the sleep duration of the KEY group was statistically significant (*p <* 0.0001). Compared with the PCPA-induced Model group, the number of crossing the central lattice in the KEY group was significantly increased, and the number of grooming was significantly reduced (all *p* < 0.05), suggesting that the anxiety behavior of the mice was improved. In addition, this study found that the compound fermentation products could significantly increase the content of neurotransmitters such as 5-HT, GABA and Glu in the hypothalamus of mice, reduce the content of inflammatory factors such as *IL-6, IL-1β* and *TNF-α* in serum, regulate the structure of intestinal flora and increase the content of short-chain fatty acids.

**Conclusion:**

Probiotic fermentation products of germinated grains can significantly improve sleep deprivation in PCPA mice, which may be related to regulating the levels of neurotransmitters and inflammatory factors, improving the structure of intestinal flora, and increasing the content of short-chain fatty acids. This study provides new candidates and research directions for the development of natural drugs to alleviate insomnia.

## 1 Introduction

Nowadays, sleep deprivation or insomnia is becoming the second most common mental disorder affecting adults worldwide, and it is especially found among women, the elderly, people with poor mental or physical health, and irregular shift workers, with insomnia affecting nearly 27% of the world’s population (Vaccaro et al., 2020). In the past decades, studies on sleep mechanisms have focused on the regulation of sleep–wake by central neurotransmitters (Saper et al., 2005). However, the initiation and maintenance of the sleep–wake cycle is not only regulated by the central nervous system. Studies have shown that there are differences in the intestinal environment between insomniacs and healthy people.

Neurotransmitters that can affect sleep mainly include 5-hydroxytryptamine (5-HT), γ-aminobutyric acid (GABA) (Byun et al., 2018), glutamate (Glu), acetyl choline (ACH), etc. GABA is a non-protein amino acid which widely exists in animals, plants, and microorganisms. It is produced by L-glutamic acid (Heli et al., 2022). 5-HT is an inhibitory neurotransmitter present in the cerebral cortex and synapses of mammals, which mainly promotes wakefulness and inhibits rapid eye movement sleep (De Deurwaerdere and Di Giovanni, 2021). Patients with insomnia have impaired intestinal barrier function, decreased diversity (Smith et al., 2019) and abundance of intestinal flora, and changes in intestinal metabolites (short-chain fatty acids (SCFAs), anti-inflammatory and pro-inflammatory factors, etc.) (Metwaly et al., 2022; Tian et al., 2022; Feng et al., 2023).

The gastrointestinal tract is involved in various physiological and pathological processes of the body and also participates in the regulation of sleep–wake mechanisms through neural, endocrine, immune, and metabolic pathways. The gut-brain axis, especially the gut microbiota, may be a key regulator of sleep mechanisms (Li et al., 2020; Badran et al., 2023). Numerous studies suggest that the regulation of sleep by the gut microbiota is achieved through the combined effects of multiple pathways within the brain-gut axis (Titos et al., 2023). The intestinal microbiome plays a significant role in modulating host sleep through the microbial-gut-brain axis by producing metabolites and compounds with neuroactive and immunomodulatory properties, including short-chain fatty acids, secondary bile acids, and neurotransmitters. Some of these metabolites and compounds are independently referred to as promoting wakefulness (serotonin, adrenaline, dopamine, orexin, histamine, acetylcholine, cortisol) or promoting sleep (γ-aminobutyric acid, melatonin). Therefore, the intestinal microbiome, through the microbial-gut-brain axis, reciprocally regulates sleep, affecting sleep quality (Xie et al., 2022; Fenk et al., 2023; Mayeli et al., 2023).

Currently, chemical medications are primarily utilized in clinical settings to enhance sleep. However, the prolonged administration of these pharmacological sleep aids may induce adverse effects, including drug dependency, drug tolerance, and rebound insomnia. In contrast, modulating the intestinal microbiota through dietary interventions, which subsequently regulates sleep via the gut-brain axis, does not pose these risks, thereby offering a broader potential for application. Consequently, non-pharmacological approaches such as cognitive behavioral therapy and dietary supplements have garnered significant attention and should be considered important avenues for research focused on improving sleep (Haarhuis et al., 2022).

In the more than 2000-year history of traditional Chinese medicine’s treatment of insomnia, substances such as wild jujubes, sprouted grains, and grape seeds, which are considered both medicinal and edible, have been found to positively affect the treatment of insomnia.

According to traditional Chinese medicine theory, sprouted grains are believed to harmonize digestion, strengthen the spleen, and stimulate appetite. Clinically, they are widely used to regulate intestinal digestive functions. (Lei et al., 2020) Additionally, sprouted grains contain an abundance of γ-aminobutyric acid (GABA) and vitamins, which can also aid in sleep (Tian et al., 2017; Lin et al., 2020).

Similarly, wild jujubes are characterized by a neutral nature and sweet flavor, associated with the heart, liver, and gallbladder meridians. They are credited with nourishing the liver, calming the mind, soothing the nerves, and controlling perspiration (Yanan et al., 2023). These attributes replenish the yin and blood of the heart and liver, addressing symptoms such as restlessness, insomnia, palpitations, and frequent dreaming. The seed kernels of wild jujubes contain a variety of active constituents including alkaloids, flavonoids, and triterpenoid saponins (Yuhao et al., 2024). These components exhibit sedative, hypnotic, anti-anxiety, and antioxidant effects, making them suitable for treating insomnia and anxiety among other conditions (Aiting et al., 2023; Yanan et al., 2023; Yuhao et al., 2024).

Grape seeds, rich in anthocyanins and catechins, are effective in scavenging free radicals and serve as potent antioxidants. They are known to regulate blood pressure and lipids, enhance immunity, protect the brain, and offer radiation protection, among other benefits. The combination of these ingredients potentially offers a multifaceted approach to sleep regulation, thereby maximizing their therapeutic efficacy (Benbouguerra et al., 2020).

In recent years, food and nutrition scientists have gradually discovered that a variety of biological changes occur during fermentation, which may increase the nutritional value of the original substances and may also lead to the production of a series of bioactive metabolites with health benefits (Lavefve et al., 2019). Probiotics, such as *Lactobacillus plantarum* (Zhu et al., 2023), *Kombucha* (Kozyrovska et al., 2012), *Acetobacter*, and *Saccharomyces cerevisiae*, can ferment food and produce various metabolites and compounds affecting other physiological processes, including short-chain fatty acids, branched-chain amino acids (BCAAs), and the like. These substances interact with the sleep regulation network in the brain through the blood circulation system and the vagus nerve afferent pathway, triggering brain arousal (Wang et al., 2022).

This study inoculates *Kombucha, Acetobacter, Saccharomyces cerevisiae, and Lactobacillus plantarum* into a microbial fermentation medium composed of jujube (Zhao et al., 2008), grain sprouts, grape seeds (Tyagi et al., 2013; Han et al., 2020), at a certain ratio. The goal is to obtain a green fermented product, KFY, which can improve sleep and healthy. This would provide a rationale for the use of natural drugs to alleviate insomnia.

## 2 Results

### 2.1 Quantification of GABA and L-theanine in the compound fermentation

A grain culture medium (Germinated millet, grape-seed powder and jujube powder were mixed at a ratio of 12:3:15, m/m), with an optimized solid-to-liquid ratio of 1:30 (w/v), was inoculated using a defined strain consortium. This consortium comprised Angel Yeast SY at 0.2 g/L, Angel Yeast RW at 0.2 g/L, *Acetic Acid Bacteria* RW at 0.2 g/L, *Lactobacillus plantarum* broth at 40 mL/L, and *Black Tea Fungus* broth at 60 mL/L. Subsequent to inoculation, the mixture underwent static fermentation at 37°C for a duration of 7 days. The product obtained post-fermentation was designated as KFY. Utilizing the Enzyme-Linked Immunosorbent Assay (ELISA), the concentrations of GABA and its exclusive bioconvertible precursor, L-Glutamic Acid, within KFY were ascertained. The concentration of GABA was found to be 12.555 μmol/L (equivalent to 1.295 mg/L), while that of L-Glutamic Acid was measured at 0.471 mg/mL.

### 2.2 Effects of KFY on sleep behavior in mice

**Figure 1:**
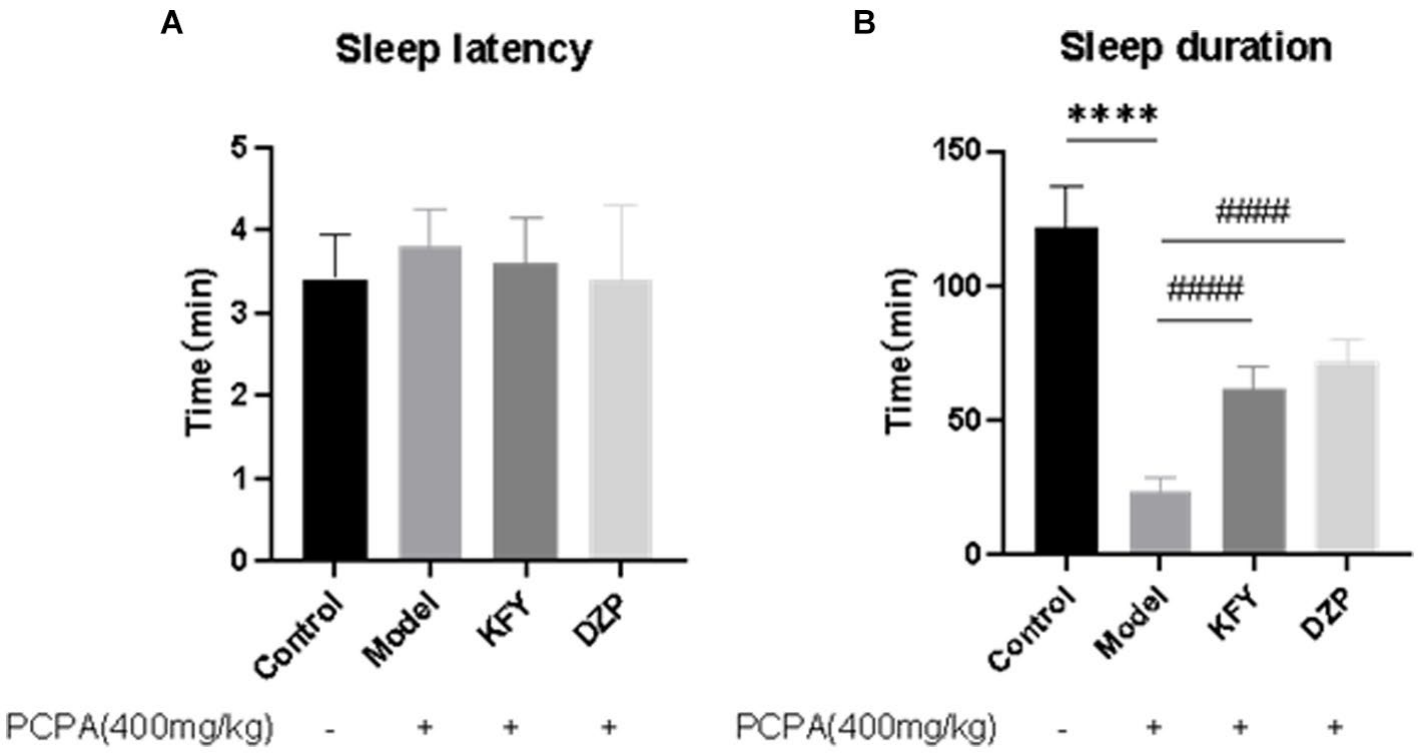
Effects of compound oral liquid on sleep behavior of PCPA induced sleep deprivation mice (*n* = 5). **(A)** Sleep latency. **(B)** Sleep duration. ****p < 0.0001; ^####^*p* < 0.0001.

After 14 days of administration, each group of mice underwent pentobarbital sodium sleep synergy experiments to observe their behavior. The change in sleep latency for each mouse was statistically analyzed using GraphPad Prism 8.3.0 software. The results showed that in the Control group, Model group, KFY group, and DZP group, Sleep latency in mice was 3.4, 3.8, 3.6, and 3.4 min, respectively (Figure 1A). In comparison to the Control group, the sleep latency slightly increased, although this difference was not statistically significant (*p* > 0.05). Similarly, while both the KFY and DZP groups had lower sleep latencies than the Model group, these differences were also not statistically significant (*p* > 0.05), this implies that these treatment methods may have little impact on the sleep onset time of mice. Regarding sleep duration analysis (Figure 1B), the sleep duration of the Model group significantly decreased compared to the Control group, and this difference was statistically significant (*p* < 0.0001). This result indicates that PCPA leads to a significant reduction in sleep duration in mice, thus confirming the success of the model establishment. In contrast, both the KFY and DZP groups exhibited an increase in sleep duration when compared to the Model group, and these differences were also statistically significant (both *p* < 0.0001). These findings suggest that fermentation products can enhance sleep quality in mice by prolonging sleep duration.

### 2.3 Effects of KFY on emotional behavior in mice

**Figure 2:**
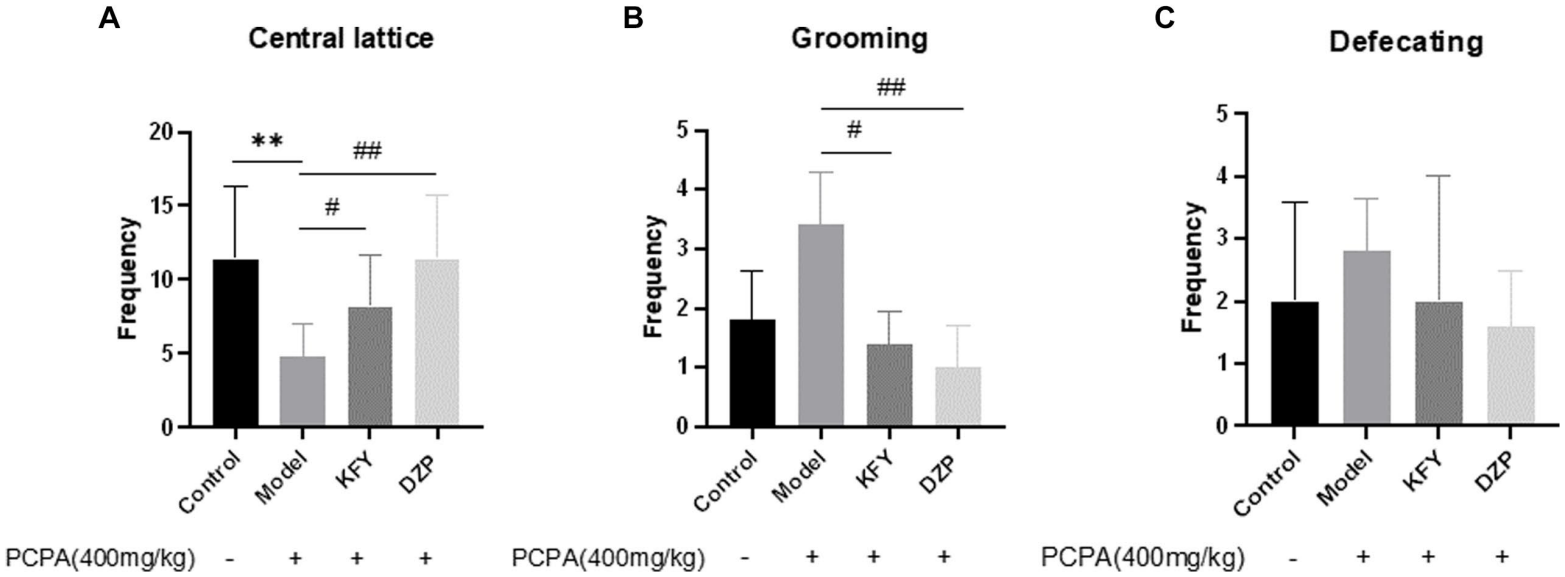
Effects of compound oral liquid on emotional behavior of PCPA-induced sleep deprivation mice (*n* = 5). **(A)** number of crossings through the central grid. **(B)** grooming times. **(C)** number of bowel movements. **p < 0.01; ^#^*p* < 0.05; ^##^*p* < 0.01.

After 14 days of administration, the results of the open field test showed that, compared to the Control group mice, the Model group mice exhibited a significant decrease in the number of times they crossed the central grid (Figure 2), indicating that PCPA-induced insomnia induced (*p* < 0.01) anxiety behavior in mice (Figure 2A). In contrast, both the KFY and DZP groups showed a significant increase in the number of times they crossed the central grid compared to the Model group (*p* < 0.05 and *p* < 0.01, respectively), suggesting that these treatments may improve anxiety behavior in mice. Moreover, the Model group mice had an increase in grooming frequency compared to the Control group. However, both the KFY (*p* < 0.05) and DZP (*p* < 0.01) groups significantly reduced the number of grooming sessions compared to the Model group (Figure 2B). Additionally, there was no significant difference in bowel movements between the Control and Model groups (*p* > 0.05). Nevertheless, both the KFY and DZP groups of mice showed a decrease in bowel movements compared to the Model group (*p* > 0.05; Figure 2C). Groups of data are expressed as a mean + standard deviation, using GraphPad Prism 8.3.0 statistical analysis software, comparing with analysis of variance, and comparing two further using Tukey ‘s multiple comparisons test.

The results indicate that PCPA sleep deprivation leads to anxiety in mice, and fermentation products KFY not only improve sleep quality but also alleviate anxiety symptoms in mice.

### 2.4 Effect of KFY on hypothalamic neurotransmitters in mice

**Figure 3:**
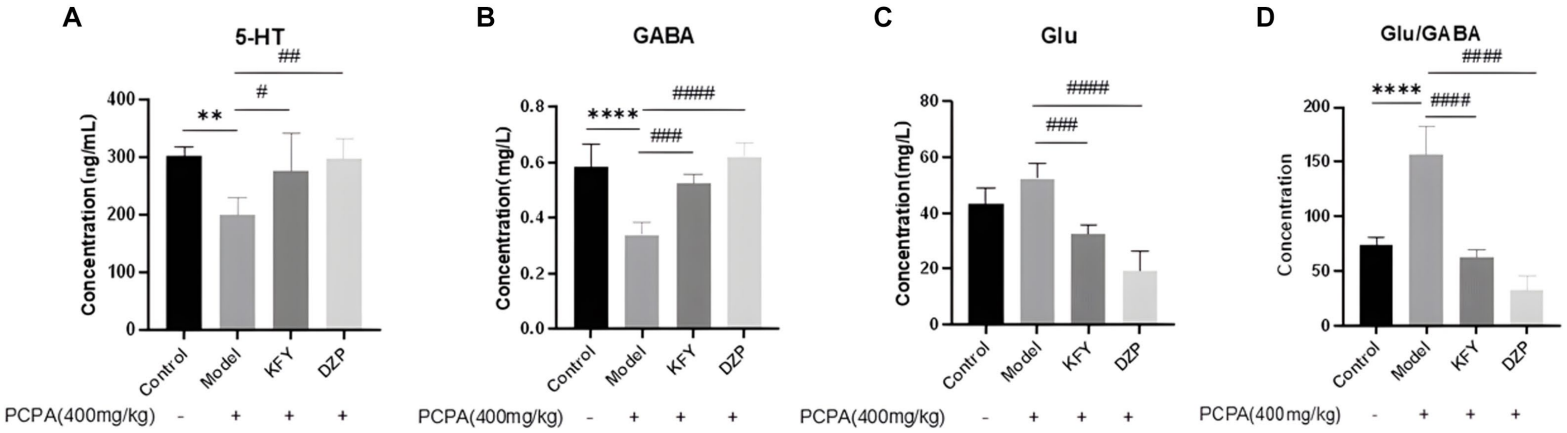
Effects of administration on neurotransmitters in hypothalamus of sleep deprived mice induced by PCPA (*n* = 5). **(A)** 5-HT. **(B)** GABA. **(C)** Glu. **(D)** Glu/GABA ratio. **p < 0.01; ****p < 0.0001; ^#^*p* < 0.05; ^##^*p* < 0.01; ^###^*p* < 0.001; ^####^*p* < 0.0001.

The data presented in Figure 3 demonstrate a significant reduction in the level of 5-HT within the hypothalamus of the Model group, compared to the Control group (*p* < 0.01), suggesting an inhibitory effect of PCPA on 5-HT synthesis. This observation is countered by the KFY and DZP groups, which exhibit a substantial recovery in 5-HT levels (*p* < 0.05 and *p* < 0.01, respectively), indicating that germinated millet fermentation product may augment 5-HT content in mice with insomnia. Parallel findings are observed in the analysis of GABA content, as shown in Figure 3B. The Model group displays a marked decrease in GABA levels relative to the Control group (*p* < 0.0001). Conversely, both the KFY and DZP groups show a notable restoration of GABA levels (*p* < 0.001 and *p* < 0.0001, respectively), pointing toward the potential of germinated millet fermentation product to enhance GABAergic neurotransmission in the hypothalamus of mice affected by insomnia. Compared with the Control group, the hypothalamus Glu level in the Model group increased, but there was no statistical significance, as depicted in Figure 3C. Nevertheless, the KFY and DZP treatments elicited a significant recovery in Glu levels (*p* < 0.001 and *p* < 0.0001, respectively), suggesting that germinated millet fermentation product may modulate glutamatergic activity in insomnia. Notably, the DZP group’s recovery in Glu levels is less pronounced than that seen with KFY. Additionally, the Glu/GABA ratio within the hypothalamus is significantly elevated in the Model group (*p* < 0.0001), as illustrated in Figure 3D. Both KFY and DZP treatments facilitate a restoration of this ratio (*p* < 0.0001), albeit without normalization. These findings imply that PCPA perturbs the balance between excitatory and inhibitory neurotransmission, potentially leading to heightened arousal. Germinated millet fermentation product appear capable of recalibrating this imbalance, promoting a more balanced neurochemical environment. Groups of data are expressed as a mean + standard deviation, using GraphPad Prism 8.3.0 statistical analysis software, comparing with analysis of variance, and comparing two further using Tukey ‘s multiple comparisons test.

### 2.5 Effect of KFY on serum inflammatory factors in mice

**Figure 4:**
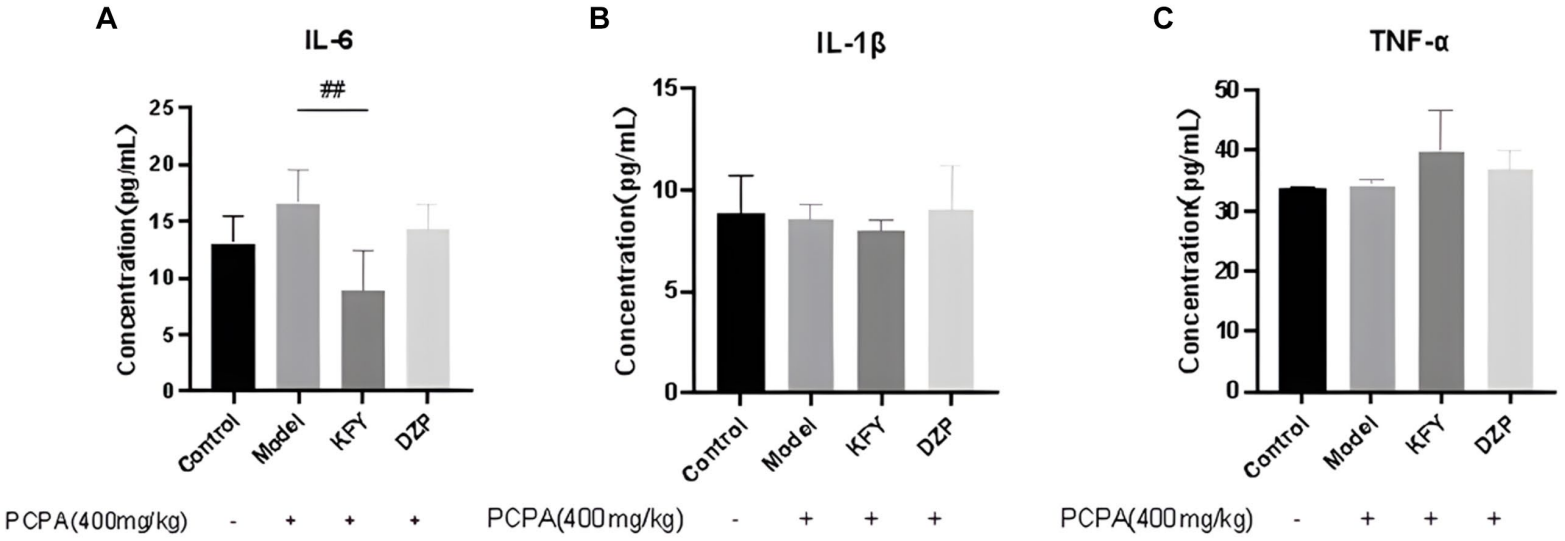
Effects of compound oral liquid on inflammatory factors in mice (*n* = 5). **(A)** IL - 6. **(B)** IL-1β. **(C)** TNF-α. ^##^*p* < 0.01.

Expression levels of inflammatory cytokines IL-6, IL-1β, and TNF-α were evaluated in mice from the Control group, model group, oral KFY group, and DZP group. The results depicted in Figure 4 demonstrate that after a 14-day treatment period, the IL-6 level in the KFY group was significantly lower than that in the model group (*p* < 0.01). In comparison to the model group, the DZP group also exhibited a reduction in IL-6 levels (*p* > 0.05), though this decrease was not statistically significant when compared to the KFY group. These findings suggest that KFY is capable of reducing IL-6 levels in mice (Figure 4A). However, for IL-1β levels across groups (Control group - 8.80 pg./mL, model group - 8.53 pg./mL, KFY group - 7.99 pg./mL, DZP group - 9.07 pg./mL), there were no statistically significant differences (*p* > 0.05; Figure 4B), indicating that treatments such as KFY have a minimal impact on IL-1β expression levels in the mouse model. Similarly, the statistical analysis of changes in TNF-α levels (Control group - 33.66 pg./mL, model group - 34.16 pg./mL, KFY group - 39.99 pg./mL, DZP group - 36.99 pg./mL) revealed no significant differences between groups (*p* > 0.05; Figure 4C), suggesting that treatments including KFY have a limited effect on TNF-α expression levels in the mouse model.

### 2.6 Effects of KFY on intestinal microbiota in mice

**Figure 5:**
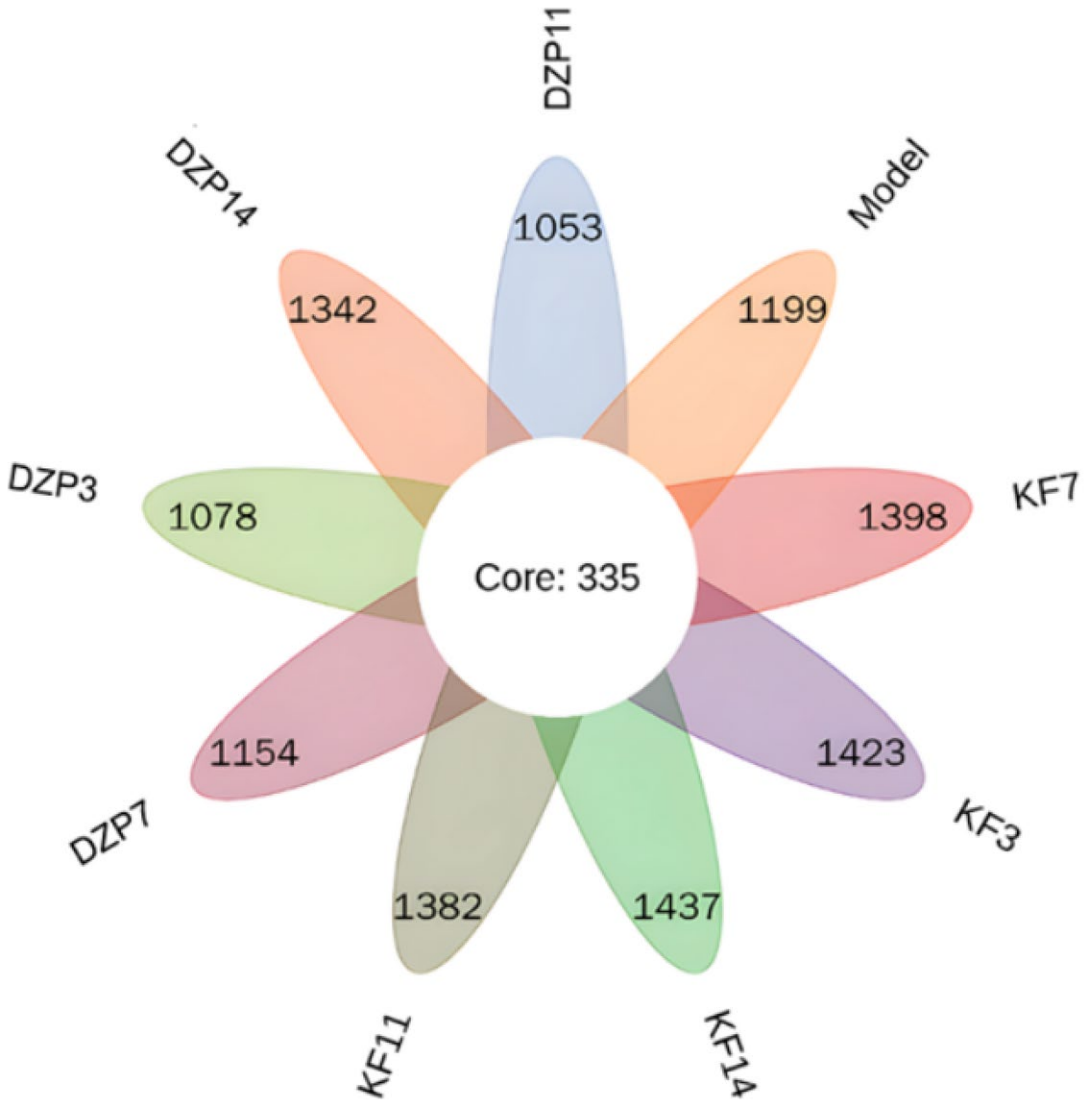
Petal diagram of intestinal flora distribution.

Special OTUs clustering analysis results are shown in Figure 5, a total of 335 between all groups OTUs. Compared with the Control group, there were 1,423, 1,398, 1,382 and 1,437 unique species in KFY3, KFY7, KFY11 and KFY14 groups, respectively, while there were 1,078, 1,154, 1,053 and 1,342 unique species in DZP3, DZP7, DZP11 and DZP14 mice treated with diazepam. Increase in the number of types of intestinal flora in mice, suggesting KFY for PCPA model mice induced by intestinal flora diversity has a regulatory role.

**Figure 6:**
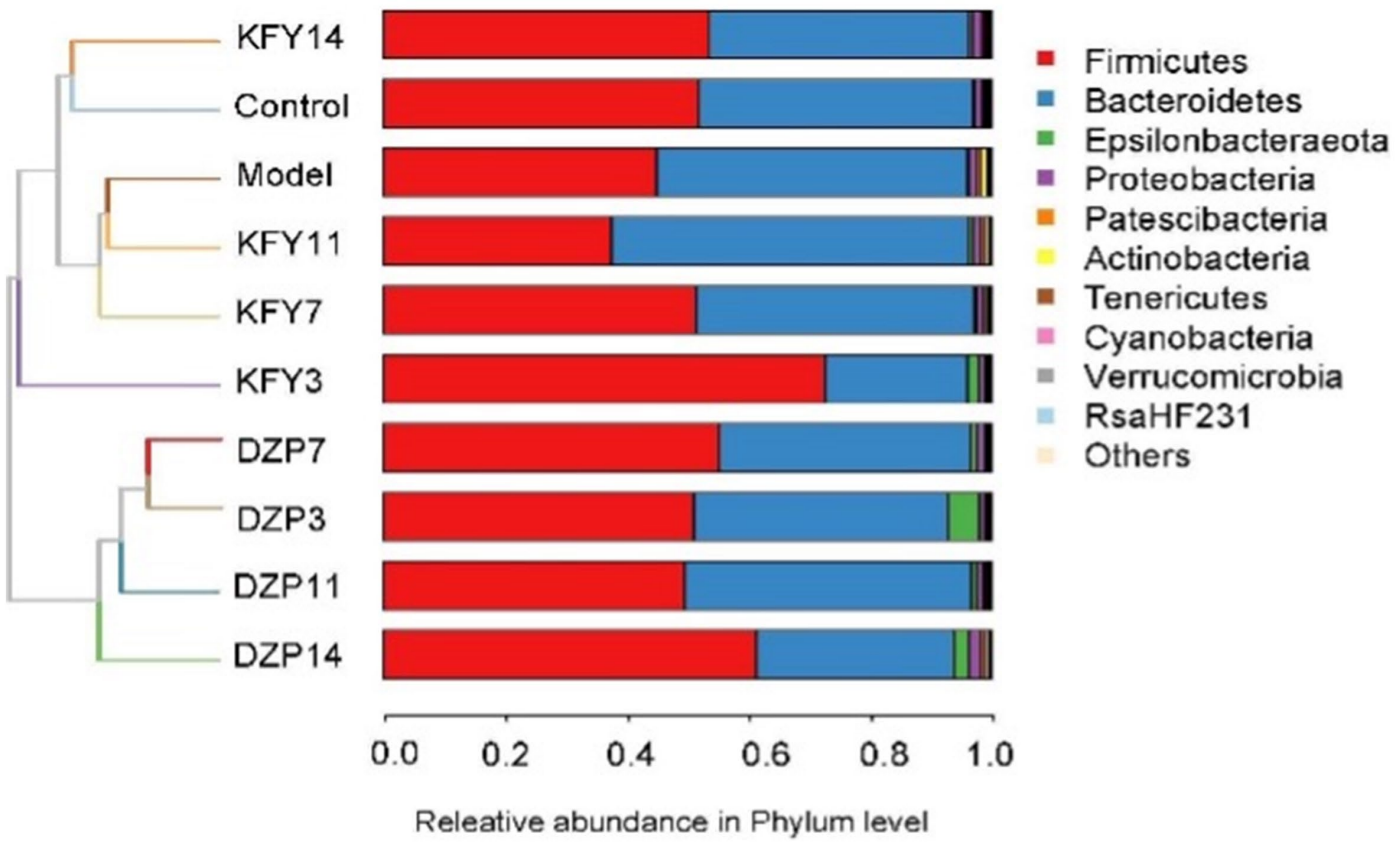
Weighted UniFrac analysis of the impact of different treatments on the microbial community structure in mouse gut.

Weighted UniFrac, an enhanced version of the UniFrac method, integrates branch lengths from the phylogenetic tree into the calculation of UniFrac distances, thereby assigning varying weights to microorganisms. This approach results in a more nuanced contribution from microorganisms with longer branch lengths to the overall UniFrac distance, thus providing a more precise representation of the differences in microbial community structure among samples. The impact of diverse treatments on the gut microbial community structure of mice was analyzed at the phylum level using Weighted UniFrac (Figure 6). It was observed that following a 14-day KFY intervention (KFY14), the gut microbiota community structure of the mice converged with that of the Control group. In contrast, mice subjected to short-term KFY interventions (e.g., KFY3, KFY7, and KFY11) exhibited gut microbiota community structures that clustered with those of the Model group. This pattern indicates that prolonged KFY intervention is capable of reversing the alterations in gut microbiota community structure induced by sleep deprivation. Concurrently, the DXP intervention groups, across various time points, formed a distinct cluster, displaying a unique microbial community structure when compared to both the control and model groups.

**Figure 7:**
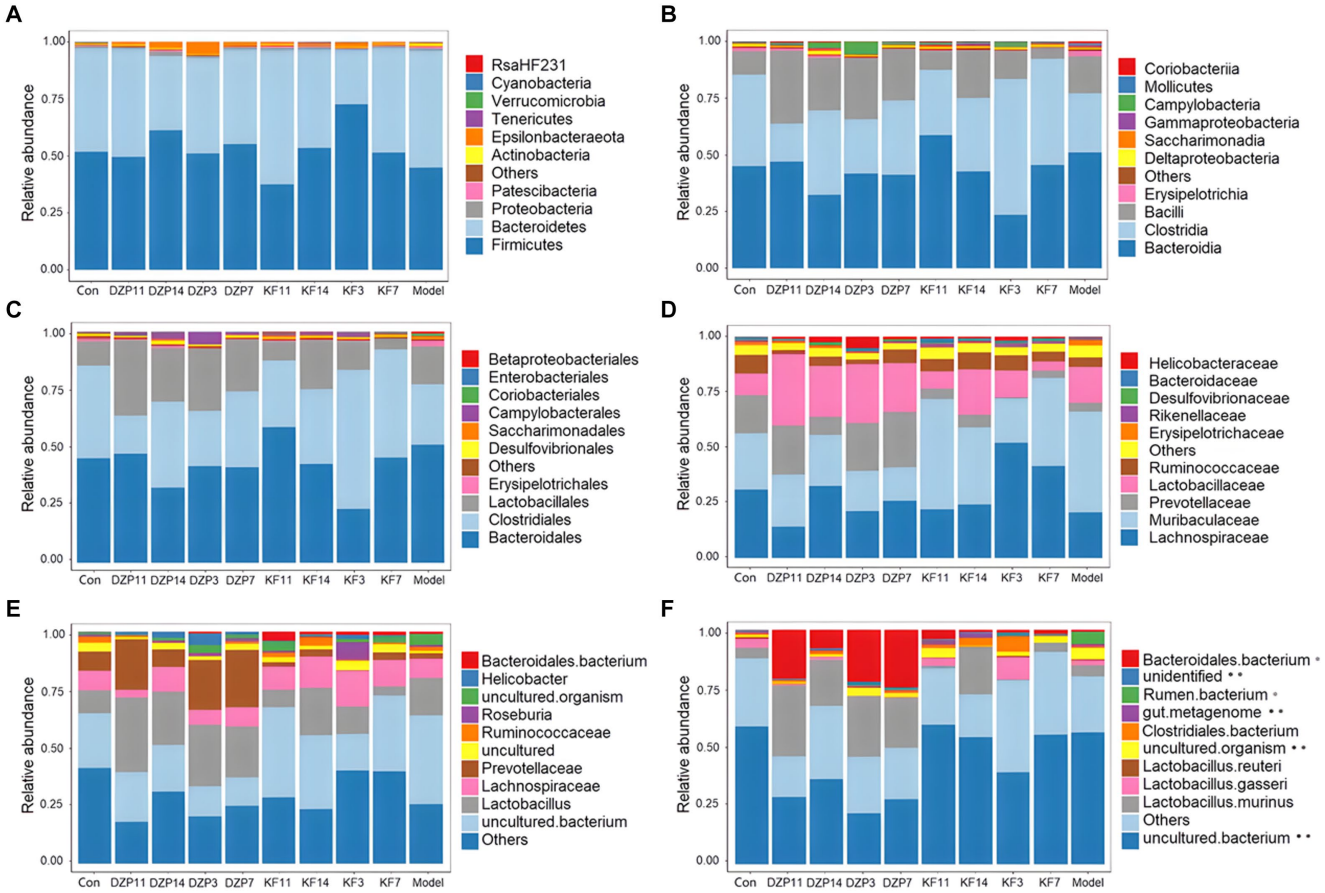
Effect of different treatments on the abundance of gut microbiota in mice. **(A)** phylum level. **(B)** class level. **(C)** order level. **(D)** family level. **(E)** genus level. **(F)** species level. KFY3, KFY7, KFY11, KFY14: mice in KFY group on 3, 7, 11, and 14 days. DZP3, DZP7, DZP11, DZP14: mice in DZP group on 3, 7, 11, 14 days. *Uncultured.

The species annotation results for each group of samples were utilized to analyze the relative abundance of species across various taxonomical levels (Phylum, Class, Order, Family, Genus, Species) in the mouse gut microbiota following different treatments. At the phylum level (Figure 7A), Bacteroidetes and *Firmicutes* predominantly characterized the intestinal community structure across all groups of mice. In comparison with the Control group, an increased relative abundance of *Actinobacteria* and *Bacteroidetes* was observed in the Model group, whereas a decrease was noted in *Firmicutes*. Conversely, when compared with the Model group, the KFY14 group demonstrated an increase in *Firmicutes* and a concomitant decrease in Actinobacteria and Bacteroidetes, with proportions of *Firmicutes* and Bacteroidetes closely resembling those in the Control group.

At the class level (Figure 7B), *Clostridia* and *Bacteroidia* primarily composed each group. Compared to the Control group, *Bacilli*, *Bacteroidia*, and Erysipelotrichia were more abundant in the Model group, whereas a reduction was seen in *Clostridia*. In contrast, the KFY14 group showed an increase in *Bacilli* and *Clostridia*, while there was a decrease in *Bacteroidia* and *Erysipelotrichia*, compared to the Model group. A 14-day KFY intervention resulted in an increase in *Clostridium* and a decrease in *Bacteroidetes* and *Erysipelothrix*, indicating that KFY could potentially ameliorate PCPA-induced changes at the class level.

Dominant flora in each group of mice at the order level (Figure 7C) were mainly *Lactobacillales*, *Clostridiales*, and *Bacteroidales*. The Model group exhibited a higher relative abundance of *Lactobacillales*, *Bacteroidales*, *Erysipelotrichales*, and *Coriobacteriales* compared to the Control group, with a corresponding decrease in *Clostridiales* and *Desulfovibrionales*. The KFY14 group displayed an increased relative abundance of *Lactobacillales* and *Clostridiales*, while *Bacteroidales*, *Erysipelotrichales,* and *Coriobacteriales* decreased. This suggests that PCPA induction led to an increase in *Lactobacillus*, *Bacteroides*, *Erysipelothrix*, and *Ruatomella*, with a decrease in *Clostridia* and *Desulfurvibrio*. However, a 14-day application of KFY resulted in an increase in *Clostridium* and a decrease in *Bacteroidetes, Erysipelothrix*, and *Ruatomella*, indicating that KFY could improve PCPA-induced alterations in bacterial flora at the order level.

At the family level (Figure 7D), compared to the Control group, the Model group showed an increased relative abundance of *Lactobacillaceae*, *Muribaculaceae*, and *Erysipelotrichaceae*, while *Prevotellaceae*, *Lachnospiraceae*, and *Ruminococcaceae* decreased. In contrast, the KFY14 group exhibited an increased relative abundance of *Ruminococcaceae*, *Lactobacillaceae*, *Pr*evotellaceae, and *Lachnospiraceae*, with a decrease in *Muribaculaceae* and *Erysipelotrichaceae*. After a 14-day KFY period post-PCPA administration, *Prevotella*, *Tricspirillaceae*, and *Ruminococcaceae* increased, while *Muribaculaceae* and *Erysipelotrichaceae* decreased, indicating that KFY could mitigate PCPA-induced perturbations at the family level of the microbial community.

At the genus level (Figure 7E), compared to the Control group, the Model group had an increased relative abundance of *Lactobacillus*, uncultured bacteria, and uncultured organisms, with a decrease in *Prevotellaceae UCG-001* and *Ruminococcaceae UCG-014*. Additionally, the relative abundance of uncultured organisms and uncultured bacteria decreased. Furthermore, after 14 days of KFY administration, *Lactobacillus* and *Verrucomicrobacteriaceae UCG-014* increased, while uncultured bacteria and uncultured organisms decreased. These findings suggest that KFY could mitigate some of the changes in genus-level microbiota induced by PCPA.

Finally, at the species level (Figure 7F), compared to the Control group, the Model group had an increased relative abundance of uncultured *rumen bacteria* and uncultured organisms, while *Lactobacillus gasseri* and *Lactobacillus murinus* decreased. When comparing the KFY14 group to the Model group, the relative abundance of uncultured *Clostridiales bacteria* and *Lactobacillus murinus* increased, while uncultured *rumen bacteria* and uncultured organisms decreased. After 14 days of KFY administration, these results suggest that KFY could alleviate some of the changes in bacterial flora at the species level caused by PCPA and induce an increase in the abundance of more unknown species.

### 2.7 Effect of drugs on intestinal SCFAs metabolism in PCPA mice

**Figure 8:**
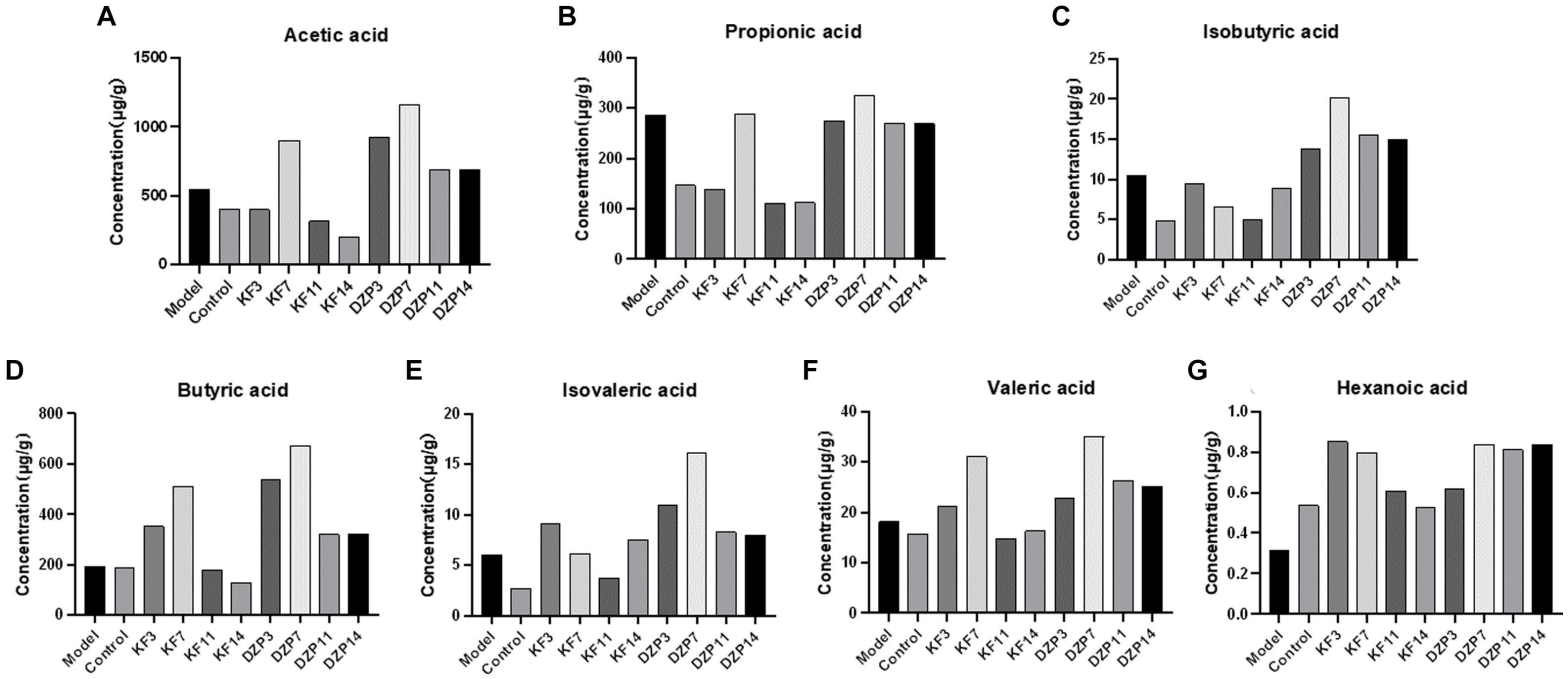
Effect of compound oral liquid on intestinal short-chain fatty acid content in mice. **(A)** acetic acid. **(B)** propionic acid. **(C)** isobutyric acid. **(D)** butyric acid. **(E)** isovaleric acid. **(F)** valeric acid. **(G)** caproic acid. KFY3, KFY7, KFY11, KFY14: mice in KFY group on 3, 7, 11, and 14 days. DZP3, DZP7, DZP11, DZP14: mice in DZP group on 3, 7, 11, 14 days.

The content of SCFAs in the feces of mice groups after intervention with different drugs was analyzed. The feces of each mouse in the same group on the same day were collected and tested for mean values. The results showed that before the establishment of the sleep deprivation model, acetic acid content was the highest, followed by butyric acid and propionic acid (Figure 8). After PCPA induction, acetic acid content remained the highest, followed by propionic acid and butyric acid. However, propionic acid content increased significantly, indicating a strong association between high levels of propionic acid concentration and lower sleep quality. After 14 days of KFY, changes were observed in all seven SCFAs, suggesting that fermentation influenced the flora producing each SCFA.

## 3 Discussion

In this study, a 14-day intervention with KFY had minimal impact on sleep latency in mice but significantly extended their sleep duration and alleviated mental anxiety induced by sleep deprivation. Since GABA, 5-HT, and Glu directly affect sleep (Yu et al., 2019), these indicators were measured, revealing that a 14-day KFY intervention increased the levels of GABA and 5-HT while reducing Glu and the Glu/GABA ratio in mice. This suggests that KFY may improve sleep by modulating neurotransmitters in the central nervous system. Therefore, we may conclude that daily intake of a certain amount of compound fermentation can reduce sleep latency and fall asleep faster, increase sleep duration, and ensure good sleep quality. In addition, compound oral liquid can not only regulate the level of 5-HT, but also improve the levels of GABA and Glu. Whether there is some relationship and change between the three needs to be explored. But we are certain that the compound fermentation in this study can improve sleep and regulate the levels of 5-HT, GABA, and Glu in the central nervous system. Therefore, this study highlights the potential of KFY’s direct regulation of hypothalamus neurotransmitters or indirect regulation through the microbiota-gut-brain axis to modulate neurotransmitters, warranting further exploration (Feng et al., 2021; Wang et al., 2022).

There is increasing evidence that insomnia is a risk factor for many diseases, including common infections and autoimmune diseases. Levels of inflammatory markers also appear to be abnormal in sleep-deprived individuals, potentially leading to low-grade inflammation. In general, pro-inflammatory factors increase the duration of Non-rapid eye movement (NREM) sleep, whereas anti-inflammatory factors have the opposite effect in animal models (Besedovsky et al., 2019). We also analyzed the effect of a 14-day KFY intervention on inflammatory factors such as IL-6, and the results suggest that KFY is capable of reducing IL-6 levels in mice, although KFY had a limited effect on TNF-α expression levels in the mouse model. The reasons involved remain unclear and necessitate further research to elucidate the relationship between KFY intervention and inflammatory factors. Elevated levels of proinflammatory cytokines (IL-1α, IL-1β, TNF-α, and IL-6) have been shown in rats subjected to 72 h of Rapid eye movement (REM) sleep deprivation. The levels of IL-1α, IL-1β, TNF-α, and IL-6 remained elevated 7 days after the experiment (Yehuda et al., 2009). These results suggest that some of the inflammatory effects of sleep deprivation may be long-lasting and may not be easily reversible. However, in the present study, serum IL-1β content decreased and IL-6 and TNF-α content increased, and these three inflammatory factors changed less significantly before and after sleep deprivation. It may be more obvious and intuitive to detect the related indicators in the hypothalamus.

Neurotransmitters such as GABA, 5-HT, and Glu, as well as levels of inflammatory factors, have a close relationship with the intestinal microbiota and their metabolites. (Mazzoli et al., 2016; Strandwitz et al., 2019). Studies have shown that healthy individuals exhibit an increase in the abundance of Firmicutes and Proteobacteria, Lachnospiraceae, whereas insomnia patients demonstrate a reduced ratio of Firmicutes to Bacteroidetes, Ruminococcaceae, and Verrucomicrobiaceae (Smith et al., 2019; Li et al., 2020; Heli et al., 2022). These findings also indicate a negative correlation with sleep deprivation. Utilizing 16S rRNA amplicon sequencing, Weighted UniFrac analysis was conducted on the gut microbiota structure of mice following various durations of KFY intervention. At the phylum level, a 14-day KFY intervention in sleep-deprived mice led to an increase in Firmicutes and a concomitant decrease in Actinobacteria and Bacteroidetes, with proportions of Firmicutes and Bacteroidetes closely resembling those in the Control group.

Numerous studies (Bishehsari et al., 2020; Frazier and Chang, 2020) have shown that *Bifidobacterium*, *Lactobacillus*, *Oscillospira*, *Akkermansia muciniphila*, *Bifidobacterium longum*, Lactobacillus, *Ruminococcus gnavus*, and Roseburia in the gut microbiota are closely associated with improved sleep quality. Bacteria in the gut microbiota, such as *Bifidobacterium* (producing acetate and lactate), *Lactobacillus* (producing lactate), *Faecalibacterium prausnitzii*, and *Roseburia* (producing butyrate), *Eubacterium* (producing butyrate and caproate), *Ruminococcus* (producing acetate, propionate, and butyrate), and Clostridium (producing butyrate), which are short-chain fatty acid-producing bacteria, can generate short-chain fatty acids that facilitate the secretion of sleep hormones, thereby enhancing sleep quality. After 14 days of KFY, changes were observed in all seven SCFAs, suggesting that fermentation influenced the flora producing each SCFA. However, further research is required to confirm how KFY affects the composition and content of SCFAs through its impact on the gut microbiota (such as fecal microbiota transplantation). Butyrate is the basic energy source for colonic epithelial cells, which is beneficial to intestinal barrier function. It is also a potential regulator of circadian clocks and metabolism in the brain and liver (Ojeda et al., 2016). It has been found that tributoryl ester, as a butyrate prodrug, improves NREM sleep in rats mediated by the vagus nerve in the portal vein area (Szentirmai et al., 2019). In addition, studies have shown that higher fecal concentrations of propionate are associated with longer periods of uninterrupted sleep in infants (Heath et al., 2020), and higher levels of propionate are associated with lower sleep efficiency in older adults with insomnia (Magzal et al., 2021). The significant increase in propionic acid after PCPA induction in the present study can prove that higher levels of propionic acid are associated with lower sleep quality. For SCFAs regulating sleep underlying mechanism is unclear, we may through the detection of two kinds of daytime and nighttime feces SCFAs content further to explore the mechanism of insomnia SCFAs intervention. At the class level, there was an increase in Bacilli and Clostridia, while there was a decrease in Bacteroidia and Erysipelotrichia after the 14-day KFY intervention in sleep-deprived mice. At the order level, there was an increased relative abundance of Lactobacillales and Clostridiales, while Bacteroidales, Erysipelotrichales, and Coriobacteriales decreased. At the family level, there was an increased relative abundance of Ruminococcaceae, Lactobacillaceae, Prevotellaceae, and Lachnospiraceae, with a decrease in Muribaculaceae and Erysipelotrichaceae. At the genus level, there was an increase in Lactobacillus and Verrucomicrobacteriaceae UCG-014, while uncultured bacteria and uncultured organisms decreased. At the species level, there was an increase in uncultured Clostridiales bacteria and *Lactobacillus murinus*, while uncultured rumen bacteria and uncultured organisms decreased. These results align with previous research, indicating that a 14-day KFY intervention may enhance sleep quality by adjusting the gut microbiota structure, promoting the proliferation of probiotics beneficial for sleep and suppressing strains detrimental to sleep.

## 4 Conclusion

In this study, we investigated the effects of KFY (Probiotic-Fermented Germinated complex) on PCPA-induced sleep deprivation in mice. Our findings indicate that KFY can increase sleep duration and alleviate anxiety behavior. This change may be due to a reduction in Glu levels and regulation of sleep–wake imbalance by supplementing hypothalamic GABA and 5-HT. Furthermore, KFY ameliorates dysbacteriosis induced by sleep deprivation by increasing the abundance of beneficial bacteria, reducing the insomnia marker bacteria.

## 5 Materials and methods

### 5.1 Strains, animals, and reagents

*Lactobacillus plantarum L9* (CGMCC 1.2469) is a proprietary strain of our research group. Kombucha was purchased from Shandong Zhi’s Weibao Biotechnology Co., Ltd. *Bacillus licheniformis* (S20010022) is a viable capsule obtained from Shenyang First Pharmaceutical Co., Ltd. of Northeast Pharmaceutical Group. The yeast used in the experiment was purchased from Angel Yeast Co., Ltd. and *Acetobacter pasteurianus* AS 1.41 (CGMCC 1.41) were obtained from Jining Yuyuan Biotechnology Co., Ltd.

The C57BL/6 female mice (7–8 weeks old, weighing 20 ± 2 g) were purchased from Jiangsu Huachuang Sino Pharmaceutical Technology Co., Ltd. (Animal license number: SCXK (Su) 2020–0009). During the experiment, all mice were housed in the animal experimental center of Yan’an University. The internal environment of the mouse room was set as follows: temperature 20 ± 2°C, relative humidity 60% ± 10%, day and night cycle 12 h light and 12 h dark alternation. The animals were provided with standard feed, allowed to drink water and move freely.

Animal handling was performed in accordance with the Guide for the Care and Use of Laboratory Animals for all animal experiments. All animals involved were approved by the Biological Research Ethics Committee of Yan’an University School of Medicine (NO: 2020–017) and conformed to animal research.

The biochemical drugs, cell culture medium components, antibodies, CCK-8 kit, ELISA kits, and other substances involved in the experiment without specific instructions were all obtained from Beijing Solarbio Technology Co., Ltd. Amplification primers for qPCR detection were synthesized by Sangon Bioengineering (Shanghai) Co., Ltd.

### 5.2 Preparation of probiotic-fermented germinated complex

Distilled water to clean millet (Yan’an City grain storage management center), water standing for 10 min, discard floating millet on the surface. Lay the remaining millet evenly in the cultivation grid in the pressing plate of the germinator, add water to the bottom of the germinator to the water level, and start the germinator to start sprouting. Germination was terminated after 72 h, during which the water was changed once a day. Germinated millet, grape-seed powder (Xunweixuan Food Manufacturing Co., LTD., Danyang City), jujube powder (Shude Agricultural Science and Technology Co., LTD., Shanxi Province), and distilled water were mixed at a ratio of 12:3:15:900. The mixture was heated at 85°C for 15 min to obtain the grain fermentation medium. 6 mL of *Lactobacillus plantarum L9* (available in our laboratory) was transferred to 100 mL of grain medium and cultured at 37°C for 7 days to obtain bacterial solution. 6 mL of *Lactobacillus plantus* preserved in glycerol was transferred to 100 mL grain medium and cultured at 37°C for 3 to 4 days to obtain bacterial solution. *Bacillus licheniformis was inoculated* on LB inoculation on solid medium, 37°C incubator for the night train, pick a single colony vaccination to 100 mL LB liquid medium, 150 rpm, 37°C oscillation training for 6 h. Take 2% inoculation based grain medium to 200 mL, 37°Cincubator 24 h. According to the instructions of the black tea fungus package (Shandong Zhishi Weibao Biological Technology Co., LTD.), 5 g black tea, 5 g green tea, 30 g white granula sugar and 1 L distilled water were weighed and placed in a clean Chinese medicine decoction machine to prepare sugar tea water. Then, the black tea fungus, Bacterial stock solution and bacterial film were added into the fermentation bottle according to the instructions. And the cooling to cool sugar tea evenly divided into the fermentation bottle, stir evenly, until the bacteria fully dissolved. The fermentation bottle was sealed with a clean square, without tightening the cap, and fermented at 25 ° C for 7 days to obtain the liquid of black tea fungus. Angel yeast (SY) and Angel yeast (RW) (Angel Yeast Co., LTD.), *Acetobacter pasteurianus AS.1.41* (Janning Yuyuan Biotechnology Co., LTD.), *Lactobacillus plantarum L9* bacterial solution (OD595 = 1), *Kombutea bacterial* solution (OD595 = 1) were added to the grain medium at a ratio of 0.02:0.02:0.02: 4 (V): 6 (V). The mixture was then fermented at 37°C for 7 days. After fermentation, the mixture was heated at 85°C for 20 min, sterilized, sealed, and stored in a refrigerator at 4°C for later use. The final fermentation product was designated KFY.

### 5.3 Determination of GABA and L-theanine content in fermented product KFY

GABA content in KFY was determined according to the instructions of the ELISA kit (Shanghai Tongwei Biotechnology Co., LTD.), and L-theanine content in KFY was determined by HPLC method with reference to the standard curve of the concentration of standard materials. The average value of GABA and theanine content in fermentation products obtained from multiple previous experiments was used as the basic index for quality control. GABA and theanine contents were determined at specific time intervals using ELISA kits and HPLC, respectively, under constant formulation and fermentation conditions. HPLC parameters: Inertsil ODS-3 (4.6 mm × 150 mm, 5 μm); Flow rate: 1.0 mL/min; Injection volume:10 μL; The detection wavelength was 210 nm. The 10 mg/mL L-theanine standard solution was diluted to 5, 2.5, 1.25 and 0.625 mg/mL with sterile water, respectively, and filtered through 0.45 μm filter membrane before injection. The retention time, peak height and peak area information were recorded, and then the concentration of each standard was taken as the horizontal axis and the peak area as the vertical axis to make the standard curve. Subsequently, standards and samples were injected, and the samples were subjected to regression analysis. When the content of GABA exceeded 1.0 mg/L, the content of L-theanine reached 0.4 mg/mL, and the fermentation product tastes sweet and sour no abnormal taste, the fermentation product was judged to be qualified and could be used in subsequent experiments.

### 5.4 Grouping and treatment of experimental animals

A total of twenty female C57BL/6 mice were randomly divided into 4 groups and administered according to the regimen outlined in Table 1. The normal Control group was intraperitoneally injected with saline at 9:00–9:30 am for two consecutive days, while the other groups were intraperitoneally injected with PCPA suspension (0.4 mg/10 g body weight) once a day at the same time to establish a sleep deprivation model in mice. The Control group was the only one not subjected to sleep deprivation. The Model group, KFY group, and DZP group were all established with a sleep deprivation model, which was validated through a pentobarbital sodium sleep synergy experiment. The Control group and Model group were provided with distilled water for 14 days as their sole source of hydration, whereas the KFY group was given fermented products KFY for free consumption over a period of 14 days, with an average daily intake of 30–35 mL per group. The treatment in the DZP group involved diluting the drug with 0.9% sodium chloride injection to a final concentration of 0.025 mg/mL, which was freely consumed for 14 days while *ad libitum* feeding and access to water sources were provided. After 14 days of processing, the sleep latency and sleep duration of the mice were recorded.

**Table 1:** Treatment and grouping of mice.

Group name	Management method	Whether to build a model	Count
Normal group (Control)	Free water	No	5
Model group (Model)	Free water	Yes	5
Experimental group (KFY)	Free drink fermented liquid	Yes	5
Diazepam group (DZP)	Drinking water with diazepam	Yes	5

The calculation methods for sleep latency and sleep duration are as follows: after intraperitoneal injection of a sub-anesthetic dose of pentobarbital sodium (the minimum dose that induces sleep in 100% of animals with an appropriate duration) in mice, wait until the animals stop moving and then flip their position to supine. If the supine position lasts for more than 60 s within 30 min, it is determined that the righting reflex has disappeared and the animal has entered a sleep state. When the righting reflex first returns, immediately flip the animal back to supine. If it regains consciousness within 30 s, it is considered awake and the time when the righting reflex first returned is recorded as the end time of sleep; if it does not recover within 30 s, repeat flipping until it is determined that sleep has ended.

Sleep latency = time to enter sleep state - time of pentobarbital sodium injection. Sleep duration = end time of sleep - time to enter sleep state.

### 5.5 Open field test of mice

After 14 days of drug administration, mice in each group were placed in an open-air experimental box with length of 40 cm × width of 40 cm × height of 30 cm, white paper and transparent glue on the inner wall, and 5 × 5 squares painted on the bottom of the box. At the beginning, the mice were placed in the central grid position to adapt for one minute. Then, a camera was used to record the behavior of the mice in the open field for six minutes. After that, relevant parameters such as the number of times the mice crossed the central grid, groomed themselves, and defecated were organized based on the video recordings.

### 5.6 Detection of neurotransmitters and inflammatory factors

After the eyeball was removed, the mouse was euthanized due to dislocation. The entire brain tissue was collected and washed with precooled physiological saline. The hypothalamus of the mouse was separated and weighed. Sodium chloride injection was added in a 1:9 ratio, thoroughly homogenized, and centrifuged at 4°C and 3,000 rpm for 20 min. The supernatant was collected and analyzed for 5-HT, GABA, and Glu using an ELISA kit according to the manufacturer’s instructions. Blood was collected from the mouse by eyeball blood collection and left at room temperature for 1 h until the blood clotted. The blood was then centrifuged at 3000 rpm for 15 min at 4°C, and IL-6, IL-1β, and TNF-α were detected using their respective ELISA kits following the manufacturer’s instructions.

### 5.7 Analysis of gut microbiota

Feces of mice were collected by tail lifting method from 8:30 am to 9:00 AM before setting up the animal model, 2 days after modeling, 3 days, 7 days, 11 days and 14 days after administration. Feces of the same group were collected into an EP tube on the same day, stored at −80°C, and transported in dry ice. The 16S rRNA sequencing analysis of the gut microbiota was performed by Shanghai Applied protein technology Co., LTD. The difference of 16S rDNA V3-V4 variable region nucleic acid sequence in fecal samples was analyzed, and the fecal intestinal flora of mice in each group was analyzed by the corresponding bioinformatics software. Genomic DNA was extracted using the magnetic bead-based soil and fecal genomic DNA extraction kit, and the DNA concentration and purity were monitored on 1% agarose gel electrophoresis.

Genomic DNA was amplified using specific primers with Barcode based on the selection of the sequencing region. All PCR reactions were carried out in a 30 μL reaction with 15 μL Phusion® high-fidelity PCR premix, and PCR amplification products were identified by electrophoresis on a 2% agarose gel. The TruSeq® DNA PCR-free Sample Preparation Kit was used to prepare the Truseq ® DNA PCR-free Sample Preparation Kit library after purification of the PCR products by Gel Extraction Kit (Qiagen). After qualified by Qubit quantification and library detection, the libraries were sequenced on the Illumina NovaSeq6000 sequencer PE250 platform.

The reads at both ends of the sequence were spliced using FLASH software. At the same time, the quality of reads and the effect of merge were filtered by quality control, and the Clean Data were obtained. Sequencing analysis was performed by the UPARSE software package using the RDP classifier algorithm. Sequences with similarity ≥97% were assigned to the same OTUs, and then the representative sequences of OTUs were aligned to the corresponding reference data for species annotation. Each Alpha diversity curve was used to assess the saturation of the overall experimental microbial community detected.

### 5.8 Analysis of fecal SCFAs

SCFAs were analyzed using gas chromatography–mass spectrometry (GC–MS), conducted by Shanghai Applied protein technology Co., LTD. Samples were thawed on ice and 30 mg of each sample was resuspended in a 2 mL glass centrifuge tube. Subsequently, 900 μL of 0.5% phosphoric acid was added to the samples, which were then shaken and mixed for 2 min before being centrifuged at 14000 g for 10 min. The supernatant of 800 μL was extracted by adding an equal volume of ethyl acetate, followed by another round of shaking and mixing for 22 min and centrifugation at 14000 g for 10 min. The upper organic phase of 600 μL was mixed with 4-methylvaleric acid (final concentration of 500 μM) as an internal standard prior to being transferred into the injection bottle for GC–MS detection. The injection volume was set at 1 μL, with a split ratio of 10:1. Separation of the samples was achieved using an FFAP column (30 m × 250 μm × 0.25 μm) for GC–MS detection. The temperature program involved an initial temperature of 100°C, which was increased to 160°C at a rate of 5°C/min, followed by a further increase to 250°C at a rate of 80°C/min and maintained for 6 min. Helium was us ed. as the carrier gas at a flow rate of 1.0 mL/min. Mass Hunter software was employed to extract the chromatographic peak areas and retention times, after which a standard curve was generated to calculate the amount of SCFAs present in the samples.

## Data availability statement

The datasets presented in this study can be found in online repositories. The names of the repository/repositories and accession number(s) can be found at: https://www.ncbi.nlm.nih.gov/, SAMN39102127, SAMN39102128, SAMN39102129, SAMN39102130, SAMN39102131, SAMN39102132, SAMN39102133, SAMN39102134, SAMN39102135, SAMN39102136.

## Ethics statement

Ethical approval was not required for the studies on humans in accordance with the local legislation and institutional requirements because only commercially available established cell lines were used. The animal study was approved by Yan’an University Animal Management Committee. The study was conducted in accordance with the local legislation and institutional requirements.

## Author contributions

JC: Formal analysis, Writing – original draft. QW: Data curation, Writing – original draft. RS: Formal analysis, Writing – original draft. WL: Formal analysis, Writing – original draft. ZW: Formal analysis, Writing – original draft. MZ: Methodology, Writing – original draft. TY: Formal analysis, Methodology, Writing – original draft. JW: Supervision, Writing – review & editing. YL: Funding acquisition, Writing – review & editing. CY: Funding acquisition, Writing – review & editing.

## References

[ref1] Aiting N. Sheng L. Chao L. Hui D. Xinbo S. (2023). Ethnopharmacological investigation of semen Ziziphi Spinosae. J. Liaoning Univ. Tradit. Chin. Med. 25, 102–107. doi: 10.13194/j.issn.1673-842x.2023.08.022

[ref2] Badran M. Khalyfa A. Ericsson A. C. Puech C. McAdams Z. Bender S. B.. (2023). Gut microbiota mediate vascular dysfunction in a murine model of sleep apnoea: effect of probiotics. Eur. Respir. J. 61:2200002. doi: 10.1183/13993003.00002-2022, PMID: 36028255 PMC11556237

[ref3] Benbouguerra N. Richard T. Saucier C. Garcia F. (2020). Voltammetric behavior, Flavanol and anthocyanin contents, and antioxidant capacity of grape skins and seeds during ripening (*Vitis vinifera* var. merlot, Tannat, and Syrah). Antioxidants (Basel) 9:800. doi: 10.3390/antiox9090800, PMID: 32867242 PMC7554950

[ref9004] Besedovsky L. Lange T. Haack M. (2019). The Sleep-Immune Crosstalk in Health and Disease. Physiol Rev 99, 1325–1380. doi: 10.1152/physrev.00010.2018 30920354 PMC6689741

[ref9005] Bishehsari F. Voigt R. M. Keshavarzian A. (2020). Circadian rhythms and the gut microbiota: from the metabolic syndrome to cancer. Nat Rev Endocrinol 16, 731–739. doi: 10.1038/s41574-020-00427-4 33106657 PMC8085809

[ref4] Byun J. I. Shin Y. Y. Chung S. E. Shin W. C. (2018). Safety and efficacy of gamma-aminobutyric acid from fermented Rice germ in patients with insomnia symptoms: a randomized, double-blind trial. J. Clin. Neurol. 14, 291–295. doi: 10.3988/jcn.2018.14.3.291, PMID: 29856155 PMC6031986

[ref5] De Deurwaerdere P. Di Giovanni G. (2021). “Chapter 1-5-HT interaction with other neurotransmitters: an overview” in Progress in brain research, 5-HT interaction with other neurotransmitters: Experimental evidence and therapeutic relevance-part A. eds. Di Giovanni G. De Deurwaerdere P. (Amsterdam, Netherlands: Elsevier), 1–5. 10.1016/bs.pbr.2021.01.001 33541674

[ref6] Feng W. Yang Z. Liu Y. Chen R. Song Z. Pan G.. (2023). Gut microbiota: a new target of traditional Chinese medicine for insomnia. Biomed. Pharmacother. 160:114344. doi: 10.1016/j.biopha.2023.114344, PMID: 36738504

[ref9003] Feng Y. Fu S. Li C. Ma X. Wu Y. Chen F.. (2021). Interaction of Gut Microbiota and Brain Function in Patients With Chronic Insomnia: A Regional Homogeneity Study. Front Neurosci 15:804843. doi: 10.3389/fnins.2021.804843 35069107 PMC8766814

[ref7] Fenk L. A. Riquelme J. L. Laurent G. (2023). Interhemispheric competition during sleep. Nature 616, 312–318. doi: 10.1038/s41586-023-05827-w, PMID: 36949193 PMC10097603

[ref9006] Frazier K. Chang E. B. (2020). Intersection of the Gut Microbiome and Circadian Rhythms in Metabolism. Trends Endocrinol Metab 31, 25–36. doi: 10.1016/j.tem.2019.08.013 31677970 PMC7308175

[ref8] Haarhuis J. E. Kardinaal A. Kortman G. A. M. (2022). Probiotics, prebiotics and postbiotics for better sleep quality: a narrative review. Benef. Microbes 13, 169–182. doi: 10.3920/BM2021.0122, PMID: 35815493

[ref9] Han X. Guo J. Yin M. Liu Y. You Y. Zhan J.. (2020). Grape extract activates Brown adipose tissue through pathway involving the regulation of gut microbiota and bile acid. Mol. Nutr. Food Res. 64:e2000149. doi: 10.1002/mnfr.202000149, PMID: 32248640

[ref9010] Heath A.-L. M. Haszard J. J. Galland B. C. Lawley B. Rehrer N. J. Drummond L. N.. (2020). Association between the faecal short-chain fatty acid propionate and infant sleep. Eur J Clin Nutr 74, 1362–1365. doi: 10.1038/s41430-019-0556-0 31969698

[ref10] Heli Z. Hongyu C. Dapeng B. Yee Shin T. Yejun Z. Xi Z.. (2022). Recent advances of γ-aminobutyric acid: physiological and immunity function, enrichment, and metabolic pathway. Front. Nutr. 9:1076223. doi: 10.3389/fnut.2022.1076223, PMID: 36618705 PMC9813243

[ref11] Kozyrovska N. O. Reva O. M. Goginyan V. B. De Vera J.-P. (2012). Kombucha microbiome as a probiotic: a view from the perspective of post-genomics and synthetic ecology. Biopolym. Cell 28, 103–113. doi: 10.7124/bc.000034

[ref12] Lavefve L. Marasini D. Carbonero F. (2019). Microbial ecology of fermented vegetables and non-alcoholic drinks and current knowledge on their impact on human health. Adv. Food Nutr. Res. 87, 147–185. doi: 10.1016/bs.afnr.2018.09.001, PMID: 30678814

[ref13] Li Y. Zhang B. Zhou Y. Wang D. Liu X. Li L.. (2020). Gut microbiota changes and their relationship with inflammation in patients with acute and chronic insomnia. Nat Sci Sleep 12, 895–905. doi: 10.2147/NSS.S271927, PMID: 33177907 PMC7652227

[ref14] Lin L. Xiao K. Y. Qu L. H. Xie Y. Wang J. Xu Y. Y.. (2020). Herbal textual research of Setariae Fructus Germinatus. Chin. J. Exp. Tradit. Med. Form. 26, 197–204. doi: 10.13422/j.cnki.syfjx.20200656

[ref15] Mayeli A. Al Zoubi O. White E. J. Chappelle S. Kuplicki R. Morton A.. (2023). Parieto-occipital ERP indicators of gut mechanosensation in humans. Nat. Commun. 14:3398. doi: 10.1038/s41467-023-39058-4, PMID: 37311748 PMC10264354

[ref9002] Mazzoli R. Pessione E. (2016). The Neuro-endocrinological Role of Microbial Glutamate and GABA Signaling. Front Microbiol. 7:1934. doi: 10.3389/fmicb.2016.01934 27965654 PMC5127831

[ref9011] Magzal F. Even C. Haimov I. Agmon M. Asraf K. Shochat T.. (2021). Associations between fecal short-chain fatty acids and sleep continuity in older adults with insomnia symptoms. Sci Rep 11:4052. doi: 10.1038/s41598-021-83389-5 33603001 PMC7893161

[ref16] Metwaly A. Reitmeier S. Haller D. (2022). Microbiome risk profiles as biomarkers for inflammatory and metabolic disorders. Nat. Rev. Gastroenterol. Hepatol. 19, 383–397. doi: 10.1038/s41575-022-00581-2, PMID: 35190727

[ref9008] Ojeda P. Bobe A. Dolan K. Leone V. Martinez K. (2016). Nutritional modulation of gut microbiota - the impact on metabolic disease pathophysiology. J Nutr Biochem 28, 191–200. doi: 10.1016/j.jnutbio.2015.08.013 26372091 PMC4757500

[ref17] Saper C. B. Scammell T. E. Lu J. (2005). Hypothalamic regulation of sleep and circadian rhythms. Nature 437, 1257–1263. doi: 10.1038/nature04284 16251950

[ref18] Smith R. P. Easson C. Lyle S. M. Kapoor R. Donnelly C. P. Davidson E. J.. (2019). Gut microbiome diversity is associated with sleep physiology in humans. PLoS One 14:e0222394. doi: 10.1371/journal.pone.0222394, PMID: 31589627 PMC6779243

[ref9001] Strandwitz P. Kim K. H. Terekhova D. Liu J. K. Sharma A. Levering J.. (2019). GABA-modulating bacteria of the human gut microbiota. Nat Microbiol. 4, 396–403. doi: 10.1038/s41564-018-0307-3 30531975 PMC6384127

[ref9009] Szentirmai É. Millican N. S. Massie A. R. Kapás L. (2019). Butyrate, a metabolite of intestinal bacteria, enhances sleep. Sci Rep 9:7035. doi: 10.1038/s41598-019-43502-1 31065013 PMC6504874

[ref19] Tian Z. T. Guang Z. Y. Ying L. Qi H. L. Jie H. F. (2017). Name changes, sources, and efficacy verification of barnyard grass. Chin. J. Med. Mater. 40, 2217–2219. doi: 10.13863/j.issn1001-4454.2017.09.050

[ref20] Tian Y. Yang W. Chen G. Men C. Gu Y. Song X.. (2022). An important link between the gut microbiota and the circadian rhythm: imply for treatments of circadian rhythm sleep disorder. Food Sci. Biotechnol. 31, 155–164. doi: 10.1007/s10068-021-01015-6, PMID: 35186346 PMC8817960

[ref21] Titos I. Juginović A. Vaccaro A. Nambara K. Gorelik P. Mazor O.. (2023). A gut-secreted peptide suppresses arousability from sleep. Cell 186, 1382–1397.e21. doi: 10.1016/j.cell.2023.02.022, PMID: 36958331 PMC10216829

[ref22] Tyagi A. Raina K. Gangar S. Kaur M. Agarwal R. Agarwal C. (2013). Differential effect of grape seed extract against human non-small-cell lung cancer cells: the role of reactive oxygen species and apoptosis induction. Nutr. Cancer 65, 44–53. doi: 10.1080/01635581.2013.785003, PMID: 23682782 PMC3859370

[ref23] Vaccaro A. Kaplan Dor Y. Nambara K. Pollina E. A. Lin C. Greenberg M. E.. (2020). Sleep loss can cause death through accumulation of reactive oxygen species in the gut. Cell 181, 1307–1328.e15. doi: 10.1016/j.cell.2020.04.049, PMID: 32502393

[ref24] Wang Z. Wang Z. Lu T. Chen W. Yan W. Yuan K.. (2022). The microbiota-gut-brain axis in sleep disorders. Sleep Med. Rev. 65:101691. doi: 10.1016/j.smrv.2022.101691 36099873

[ref25] Xie Z. Zhang X. Zhao M. Huo L. Huang M. Li D.. (2022). The gut-to-brain axis for toxin-induced defensive responses. Cell 185, 4298–4316.e21. doi: 10.1016/j.cell.2022.10.001, PMID: 36323317

[ref26] Yanan Z. Mingyang T. Huimin L. Fang D. Zhenni G. Zan W. (2023). Research progress on the use of Suanzaoren decoction in treating insomnia disorders. J. Stroke Neurol. Dis. 40, 81–83. doi: 10.19845/j.cnki.zfysjjbzz.2023.0021

[ref9007] Yehuda S. Sredni B. Carasso R. L. Kenigsbuch-Sredni D. (2009). REM sleep deprivation in rats results in inflammation and interleukin-17 elevation. J Interferon Cytokine Res. 29, 393–398. doi: 10.1089/jir.2008.0080 19450150

[ref9012] Yu X. (2019). GABA and glutamate neurons in the VTA regulate sleep and wakefulness. Nat Neurosci 22, 106–119. doi: 10.1038/s41593-018-0288-9 30559475 PMC6390936

[ref27] Yuhao S. Yi N. Wei Z. Lan Y. Haizhen L. Xiaojuan C.. (2024). Qualitative and semi-quantitative analysis of the chemical composition of semen Ziziphi Spinosae pericarp and kernel. Chromatography 42, 234–244.

[ref28] Zhao Z. Liu M. Tu P. (2008). Characterization of water soluble polysaccharides from organs of Chinese jujube (*Ziziphus jujuba* mill. cv. Dongzao). Eur. Food Res. Technol. 226, 985–989. doi: 10.1007/s00217-007-0620-1

[ref29] Zhu R. Fang Y. Li H. Liu Y. Wei J. Zhang S.. (2023). Psychobiotic *Lactobacillus plantarum* JYLP-326 relieves anxiety, depression, and insomnia symptoms in test anxious college via modulating the gut microbiota and its metabolism. Front. Immunol. 14:1158137. doi: 10.3389/fimmu.2023.1158137, PMID: 37033942 PMC10077425

